# Interaction between *HTR2A* rs3125 and negative life events in suicide attempts among patients with major depressive disorder: a cross-sectional study

**DOI:** 10.1186/s12888-024-05713-3

**Published:** 2024-04-02

**Authors:** Jian-Yue Pang, Yi-Ping Wang, Hui-Min Teng, Jin He, Rui Luo, Si-Meng Feng, Wei-Hua Yue, Heng-Fen Li

**Affiliations:** 1 Department of Psychiatry, The First Affiliated Hospital of Zhengzhou University, Zhengzhou University, 450052 Zhengzhou, China; 2 Peking University Sixth Hospital, Peking University Institute of Mental Health, 100191 Beijing, China; 3 National Clinical Research Center for Mental Disorders (Peking University Sixth Hospital), 100191 Beijing, China; 4 NHC Key Laboratory of Mental Health (Peking University), 100191 Beijing, China; 5 PKU-IDG/McGovern Institute for Brain Research, Peking University, 100871 Beijing, China; 6 Chinese Institute for Brain Research, Beijing102206, China

**Keywords:** Major depressive disorder, Suicide attempts, *HTR2A*, Negative life events, Gene-environment interaction

## Abstract

**Background:**

Both genetic and environmental factors play crucial roles in the development of major depressive disorder (MDD) and suicide attempts (SA). However, the interaction between both items remains unknown. This study aims to explore the interactions between the genetic variants of the serotonin 2 A receptor (*HTR2A*) and the nitric oxide synthase 1 (*NOS1*) and environmental factors in patients who experience MDD and SA.

**Methods:**

A total of 334 patients with MDD and a history of SA (MDD-SA) were recruited alongside 518 patients with MDD with no history of SA (MDD-NSA), and 716 healthy controls (HC). The demographic data and clinical characteristics were collected. Sequenom mass spectrometry was used to detect eight tag-single nucleotide polymorphisms (tagSNPs) in *HTR2A* (rs1328683, rs17068986, and rs3125) and *NOS1* (rs1123425, rs2682826, rs3741476, rs527590, and rs7959232). Generalized multifactor dimensionality reduction (GMDR) was used to analyze the gene-environment interactions.

**Results:**

Four tagSNPs (rs17068986, rs3125, rs527590, and rs7959232) exhibited significant differences between the three groups. However, these differences were not significant between the MDD-SA and MDD-NSA groups after Bonferroni correction. A logistic regression analysis revealed that negative life events (OR = 1.495, 95%CI: 1.071–2.087, *P* = 0.018), self-guilt (OR = 2.263, 95%CI: 1.515–3.379, *P* < 0.001), and negative cognition (OR = 2.252, 95%CI: 1.264–4.013, *P* = 0.006) were all independently associated with SA in patients with MDD. Furthermore, GMDR analysis indicated a significant interaction between *HTR2A* rs3125 and negative life events. Negative life events in conjunction with the *HTR2A* rs3125 CG + GG genotype were associated with a higher SA risk in patients with MDD when compared to the absence of negative life events in conjunction with the CC genotype (OR = 2.547, 95% CI: 1.264–5.131, *P* = 0.009).

**Conclusion:**

Several risk factors and a potential interaction between *HTR2A* rs3125 and negative life events were identified in patients with SA and MDD. The observed interaction likely modulates the risk of MDD and SA, shedding light on the pathogenesis of SA in patients with MDD.

**Supplementary Information:**

The online version contains supplementary material available at 10.1186/s12888-024-05713-3.

## Introduction

Major depressive disorder (MDD) and suicide attempts (SA) are significant public health issues worldwide due to their high prevalence and incidence rates. Approximately 800,000 people die from suicide globally each year, marking a distressing trend of increasing suicide rates over the past 20 years [1–3]. Notably, over 90% of people who die from suicide are reported to have some form of mental disorder, with MDD being especially prominent [4]. Suicide is considered the most serious consequence of MDD [5, 6]. Previous studies have demonstrated that patients with MDD and a history of SA have a higher risk of dying from suicide compared to those with no history of SA [7–9]. Thus, there is an urgent need to define the risk factors and pathogenesis of SA. This effort will enable earlier identification and intervention in patients with MDD exhibiting suicidal behaviors.

Genetic factors play a crucial role in MDD [10, 11]. Population-based studies have confirmed that the heritability of MDD is 30–40% [12], with the corresponding suicide risk also having genetic characteristics [13]. Notably, studies have demonstrated that genetic susceptibility to MDD increases the risk of suicide [14, 15]. Many biological factors and candidate genes have been associated with MDD and SA [16, 17], including the serotonin 2 A receptor (*HTR2A*) gene and the nitric oxide synthase 1 (*NOS1*) gene. There is evidence indicating that the density of HTR2A is increased in brain regions of depressed suicide patients [18, 19]. Hrdina et al. observed that platelet HTR2A was significantly up-regulated in patients with MDD who have suicidal thoughts compared to patients without suicidal thoughts. They further noted that the elevated HTR2A levels persisted even after antidepressant treatment [20]. Several studies have also suggested an association between *NOS* genes and suicidal behavior [21, 22], with a lack of *NOS1* resulting in increased impulsivity, aggression, and other abnormal social behaviors. A study revealed lower NOS protein levels in the brain tissue of patients with MDD who died by suicide compared to the normal control group [23]. Additionally, lower levels of *NOS1* mRNA have been observed in the anterior cingulate cortex of patients with MDD [24, 25]. Thus, *HTR2A* and *NOS1* genes may play a crucial role in determining whether individuals with MDD may attempt suicide.

Increasing evidence supports the involvement of both heritable and environmental risk factors in MDD and SA [26–28]. Environmental factors, including early trauma and recent acute/chronic stress (separation, loss, interpersonal or family problems, poor social contacts/support, and occupational stress/unemployment), have long been recognized to play pivotal roles in both MDD development and SA [6, 29, 30]. Gene-environment investigations have consistently revealed *HTR2A* to be associated with both MDD and SA and have yielded conflicting results. Notably, rs6313 demonstrated some level of interaction with both MDD [28] and suicidal behavior [31, 32]. However, this finding was not consistent across several studies [32, 33]. A study revealed an interaction between rs6311 (–1438G > A) and a measure of familial environment in MDD [34]. Another study reported three variants (rs6561333, rs7997012, and rs1885884) of *HTR2A* that interact with early trauma in association with SA but not with MDD [35]. This inconsistency may be due to varied forms of measurement of environmental risk. However, there is currently a lack of research on the interaction between *NOS1* and environmental factors in patients with SA and MDD.

In this study, it was hypothesized that the interaction between genetic variants of *HTR2A* and *NOS1* and environmental factors plays a crucial role in the development of suicidal behavior in patients with MDD. Therefore, this study aims to explore the associations among the *HTR2A* gene, *NOS1* gene, and environmental factors in patients with MDD who have a history of SA.

## Methods

### Subjects

Patients with MDD were recruited from the First Affiliated Hospital of Zhengzhou University over the period from 2009 to 2014. The inclusion criteria were as follows: (1) meeting the diagnostic criteria for MDD as specified by the Diagnostic and Statistical Manual of Mental Disorders, Fourth Edition (DSM-IV); (2) presenting a score ≥ 21 on the Hamilton Depression Rating Scale-24 items (HDRS-24); (3) being 18 to 65 years of age; and (4) being of Han Chinese ethnicity. The following exclusion criteria were used: (1) presenting with comorbid psychotic illness; (2) having a family history of inherited diseases; (3) having organic mental disorders; and (4) having psychoactive substance abuse.

Healthy controls (HC) from the Physical Examination Center of the First Affiliated Hospital of Zhengzhou University were enrolled as the control group. Inclusion criteria for healthy controls consisted of (1) having no family history of mental disorders; (2) having an HDRS-24 score of < 7; (3) being aged 18 years to 65 years; and (4) being of Han Chinese ethnicity. Individuals with family histories of inherited diseases or psychoactive substance abuse were excluded.

A total of 852 patients with MDD and 716 HC were included in this study. SA was defined as suicidal behaviors associated with the intention to end one’s own life but not leading to death. In the clinical interview, all subjects and/or their guardians or other persons with whom they had lived for an extended period were asked “In your (or the patient’s) course of MDD, did you (he or she) ever attempt suicide?” If the participant answered “yes,” these patients were regarded as having a history of SA. Negative life events were collected by asking “Did you (he or she) experience unemployment, separation, loss, interpersonal or family problems before SA?” If they answered “yes”, negative life events are recorded as “Yes”, otherwise as “No”. Childhood trauma history was collected by asking “Did you (he or she) experience sexual or physical abuse before SA?” If they answered “yes”, childhood trauma history was recorded as “Yes”, otherwise as “No”.

To reduce recall bias, patient data were collected and verified by their guardians or other people with whom they had lived for an extended period. The patients were divided into MDD-SA and MDD-NSA groups based on whether they had a history of SA during the overall course of MDD. This study was approved by the Ethics Committee of the First Affiliated Hospital of Zhengzhou University and all participants signed an informed consent form before they participated in the study.

### Clinical data

The clinical data obtained from the study participants included sociodemographic data and clinical features. The diagnosis was confirmed using the Structured Clinical Interview for DSM-IV Axis I. Sociodemographic data were collected using a self-designed demographic questionnaire, and the items of the HDRS-24 were applied to assess symptom severity. Sociodemographic data included six variables: sex, age, marital status, childhood trauma history, family history of psychosis, and negative life events. The clinical features included 13 variables: age at onset, number of episodes, overall course of illness, depressive symptoms (including depressed mood, loss of interest, lack of energy, self-guilt, negative cognition, weight loss, circadian rhythms, waking up early, sexual dysfunction, and psychotic symptoms). Date for family history of psychosis and depressive symptoms were collected by asking “Did you have a family history of psychosis or depressive symptoms?” Based on the answer, they were recorded as “Yes” or “No”. To reduce sampling bias, the research team members were trained in clinical diagnosis, scale evaluation, and clinical interviews, and a consistency test was conducted.

### TagSNPs data

Using data from the NCBI database (HapMap Data Rel 28Phase II + III, August 10, NCBI B36 assembly, dbSNP bl26-CHB + JPT data), specific single nucleotide polymorphisms (SNPs) of *HTR2A* and *NOS1* were selected for analysis. The Haploview 4.2 software was used to select eligible tag-single nucleotide polymorphisms (tagSNPs), with minimum allele frequency (MAF) > 0.05 and r^2^ ≥ 0.8 as the selection conditions. The subjects’ DNA was extracted using TIANGEN’s DP348-3 whole blood genomic DNA extraction kit. The DNA samples were collected by silica gel membrane adsorption and subsequently genotyped. Eight tagSNPs (rs1328683, rs17068986, and rs3125 in *HTR2A* and rs1123425, rs2682826, rs3741476, rs527590, and rs7959232 in *NOS1*) were detected using Sequenom mass spectrometry (see Additional file 1). The genotypes of the eight tagSNPs in the three groups were consistent with the H-W balance law (*P* > 0.05).

### Statistical analysis

Statistical Package for the Social Sciences (SPSS version 25) was used to analyze the data. Continuous variables with normal distribution were analyzed using independent t-tests or one-way analysis between two or three groups, with results presented as mean ± standard deviation (SD). Conversely, continuous variables deviating from normal distribution were subjected to non-parametric tests, and outcomes were reported as median (interquartile range). The chi-square test was employed for qualitative variables, and Bonferroni correction was applied for multiple comparisons. Logistic regression analysis was conducted to examine the factors that significantly influenced SA in patients with MDD.

Gene-environment interactions were analyzed using the generalized multifactor dimensionality reduction (GMDR) [36] BetaV0.7 software package. Gene-environment interactions can reflect a causal mechanism where one or more environmental factors contribute to the causation of a condition in the same individual with genetic factors influencing the sensitivity to environmental exposures. The best gene-environment interaction model was selected based on the values obtained from cross-validation (CV) consistency and accuracy testing. Odds ratios (OR) were computed with a 95% confidence interval (CI) using logistic regression to determine the set of risk factors identified by the GMDR analysis. A two-sided analysis was performed, with the threshold for statistical significance set at *P* < 0.05. However, the threshold for statistical significance after Bonferroni correction was set at *P* = 0.0167.

## Results

### Sociodemographic data and clinical features

A total of 334 out of 852 patients with MDD had a history of SA (Additional file 2). The SA rate was 39.2%. There were no significant differences between the three groups in terms of mean age (F = 0.433, *P* = 0.805) or sex (χ^2^ = 4.595, *P* = 0.101). The MDD-SA and MDD-NSA groups differed significantly in terms of negative life events (χ^2^ = 8.63, *P* = 0.004), lack of energy (χ^2^ = 5.46, *P* = 0.019), self-guilt (χ^2^ = 38.58, *P* = 0.000), negative cognition (χ^2^ = 10.01, *P* = 0.002), weight loss (χ^2^ = 6.61, *P* = 0.010), and circadian rhythms (χ^2^ = 9.20, *P* = 0.003).

### Eight tagSNPs of MDD-SA vs. MDD-NSA vs. HC

Significant differences were observed in the genotype and allele frequencies of *HTR2A* rs17068986 (χ^2^ = 13.892, *P* = 0.008) and rs3125 (χ^2^ = 13.992, *P* = 0.007), as well as *NOS1* rs527590 (χ^2^ = 13.330, *P* = 0.010) and rs7959232 (χ^2^ = 13.636, *P* = 0.009) among the three groups (Additional file 3). Bonferroni correction for further pairwise comparisons revealed no statistical differences in genotype or allele frequencies between the MDD-SA and MDD-NSA groups (*P* > 0.0167). Statistical differences only existed between the MDD-SA and HC groups or the MDD-NSA and HC groups (*P* < 0.0167).

### The risk factors for SA in patients with MDD

Ten variables exhibiting statistical differences (Additional file 2 and Additional file 3) were included in the multifactor logistic regression analysis to identify the risk factors for SA in patients with MDD. Negative life events (OR = 1.495, 95% CI: 1.071–2.087, and *P* = 0.018), self-guilt (OR = 2.263, 95% CI: 1.515–3.379, and *P* = 0.000), and negative cognition (OR = 2.525, 95% CI: 1.264–4.013, and *P* = 0.006) were all independently associated with SA in patients with MDD (Table 1).

**Table 1:** Risk factors for SA in patients with MDD

Variable		Genotype	B	*P*	OR	95%CI
	*HTR2A* rs17068986	CC		reference		
		TC	0.817	0.746	1.083	0.667–1.760
		TT	0.272	0.345	1.313	0.746–2.309
	*HTR2A* rs3125	CC		reference		
		CG	-0.284	0.584	0.753	0.273–2.078
		GG	-0.775	0.136	0.461	0.166–1.275
	*NOS1* rs527590	CC		reference		
		CT	-0.054	0.769	0.948	0.663–1.356
		TT	0.335	0.200	1.398	0.838–2.334
	*NOS1* rs7959232	AA		reference		
		AG	-0.204	0.316	0.815	0.547–1.216
		GG	-0.156	0.487	0.855	0.550–1.330
	Negative life events		0.402	**0.018**	1.495	1.071–2.087
	Weight loss		0.170	0.341	1.185	0.835–1.681
	Lack of energy		0.281	0.301	1.324	0.778–2.254
	Self-guilt		0.817	**0.000**	2.263	1.515–3.379
	Negative cognition		0.812	**0.006**	2.252	1.264–4.013
	Circadian rhythms		0.135	0.426	1.144	0.821–1.595

OR, odds ratio; CI, confidence interval

### The gene-environment interaction for SA in patients with MDD

**Fig. 1:**
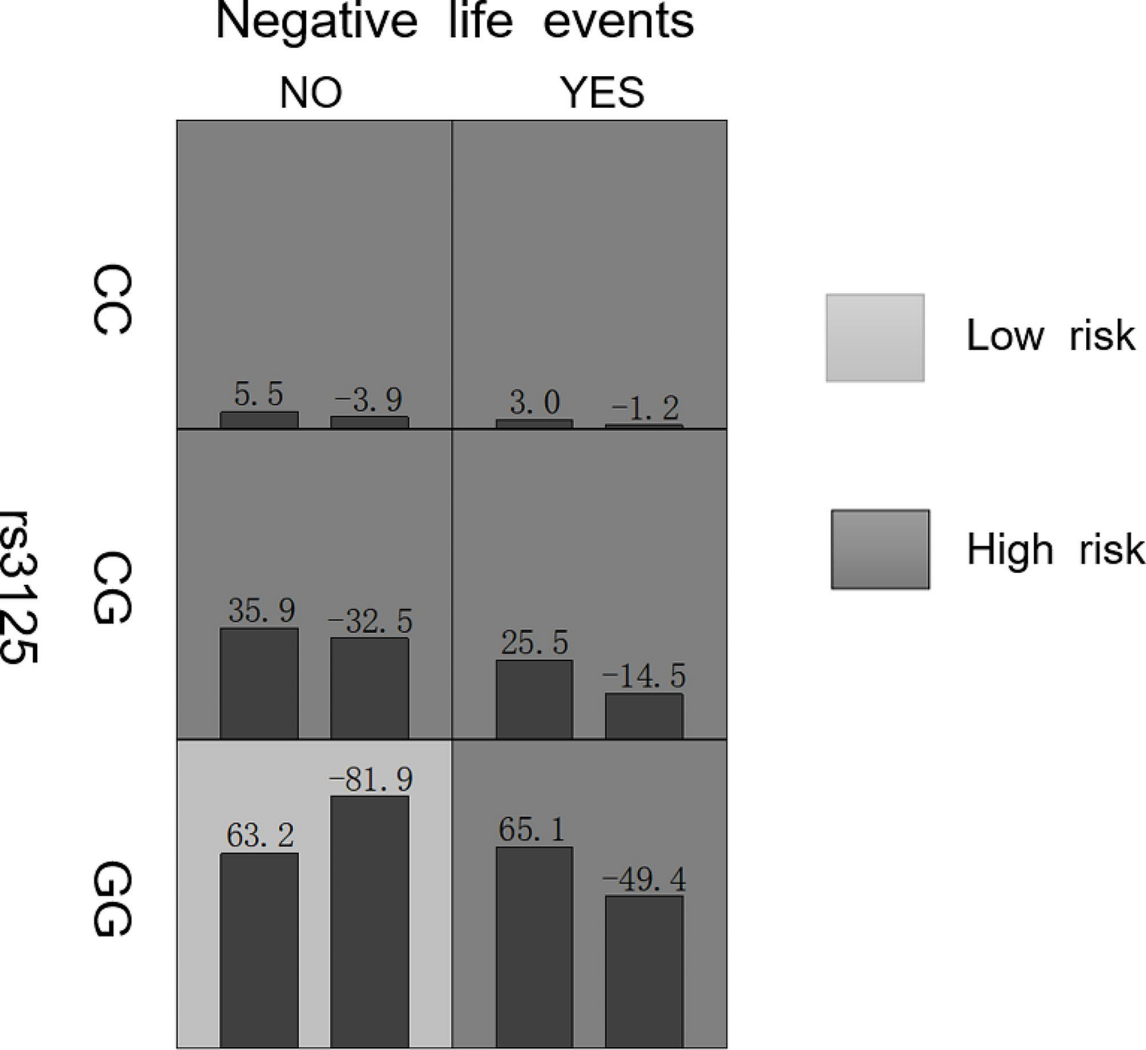
Analysis of gene-environment interactions between *HTR2A* rs3125 and negative life events using GMDR. The dark gray cell represents the high risk factors and the light gray cell represents the low risk factors. The left and right columns represent the maximum likelihood estimates of the weights for the MDD-SA and MDD-NSA groups, and are expressed as positive and negative scores. The sum of positive and negative score is used to judge the high or low risk of the combination. NO and YES denote no negative life events and have negative life events, respectively. Among them, individuals with GG genotype of rs3125 when exposed negative life events had the highest SA risk with the highest sum score. *HTR2A*, serotonin 2 A receptor; GMDR, generalized multifactor dimensionality reduction; MDD-SA group, major depressive depression with suicide attempts; MDD-NSA group, major depressive depression without suicide attempt

Four tagSNPs (*HTR2A* rs27068986 and rs3125, *NOS1* rs527590 and rs7959232) exhibiting statistical differences (Additional file 3) and environmental factors (childhood trauma history and negative life events) were incorporated into the GMDR to analyze the gene-environment interactions for SA in patients with MDD. Table 2 summarizes the results obtained from the GMDR analyses of one- to six-locus models. There was a significant two-locus model (*P* = 0.011) involving rs3125 and negative life events, indicating a potential gene-environment interaction between rs3125 and negative life events. The two-locus models had a test accuracy of 0.578 and a cross-validation consistency of 10/10. Figure 1 depicts the detailed gene-environment interactions between *HTR2A* rs3125 and negative life events. Participants with the GG genotype of rs3125 and negative life events were at a highest risk for SA with the highest sum score.

**Table 2:** Gene-environment interaction among 4 tagSNPs, childhood trauma history, and negative life events

Locus no.	Best combination	Testing accuracy	CV consistency	*P*
1	rs7959232	0.512	6/10	0.172
2	rs3125, NLE	0.578	10/10	**0.011**
3	rs3125, rs7959232, rs527590	0.514	6/10	0.055
4	rs3125, rs7959232, rs527590, NLE	0.498	6/10	0.828
5	rs3125, rs17068986, rs7959232, rs527590, NLE	0.486	10/10	0.945
6	rs3125, rs17068986, rs7959232, rs527590, NLE, CTH	0.496	10/10	0.828

NLE, negative life events; CTH, childhood trauma history; CV: cross-validation

OR and 95% CI were obtained for the set of risk factors identified from the GMDR analysis to further assess the identified gene-environment interactions associated with SA in MDD. To narrow down the number of possible combinations, we used the combination of CC genotype of rs3125 and non-negative events as the reference group. Notably, negative life events, together with the CG + GG genotype were significantly associated with SA in patients with MDD compared to non-negative life events for those with the CC genotype (OR = 2.547, 95% CI: 1.264–5.131, *P* = 0.009) (Table 3).

**Table 3:** Interaction between *HTR2A* rs3125 genotype and negative life events for SA in patients with MDD

HTR2A rs3125	negative life events	B	*P*	OR	95% CI
CC	NO		reference		
CC	YES	0.377	0.284	1.459	0.731–2.909
CG + GG	NO	-0.151	0.762	0.860	0.325–2.278
CG + GG	YES	0.935	**0.009**	2.547	1.264–5.131

OR, odds ratio; CI, confidence interval

## Discussion

In this study, we investigated the risk factors and interaction of polymorphisms in two genes, along with environmental factors in relation to SA in patients with MDD. Three risk factors were identified for SA in patients with MDD: negative life events, self-guilt, and negative cognition. Two-locus models were also found between *HTR2A* rs3125 and negative life events in relation to SA in patients with MDD. The combination of negative life events and *HTR2A* rs3125 CG + GG genotype was associated with a higher SA risk in patients with MDD. These findings are consistent with those of previous studies indicating that genes affect the development of suicidal behavior and MDD not only through direct effects on MDD risk but also by modulating individuals’ sensitivity to environmental factors.

Both MDD and SA have genetic predispositions. Several studies have suggested a relationship between *HTR2A* and *NOS1* genes and SA in patients with MDD [35, 37–41]. However, the results obtained have been inconsistent. This present study revealed significant differences in the four tagSNPs—rs17068986 and rs3125 of *HTR2A*, as well as rs527590 and rs7959232 of *NOS1—*across the MDD-SA, MDD-NSA, and HC groups. However, none of the associations retained statistical significance upon applying the Bonferroni correction test. Consistent with the results of this study, previous studies also reported no association between the tagSNPs of the *HTR2A* gene and SA in patients with MDD [42–45]. A study involving French adolescents suggested that rs2682826 of *NOS1* was not associated with SA [46], consistent with the results of our study. However, several studies have reported inconsistent results in this area [40, 47, 48]. A study in Japan revealed that rs2682826 of *NOS1* was associated with completed suicides, especially in male patients [48]. However, the association between genes and SA in patients is inconsistent. A major reason for these inconsistent findings is that both MDD and SA are complex, heterogeneous diseases caused by a combination of multiple gene variants, each of which has only a small impact on disease risk and symptoms.

This study also revealed that symptoms of self-guilt, negative cognition, and negative life events were associated with increased suicide risk in patients with MDD, consistent with the findings of previous studies [49–51]. In a multi-ethnic Asian population study, patients with repeated SA reported having experienced a greater level of adverse life events such as unemployment, divorce, quarrels, and negative emotions [49]. A meta-analysis revealed that SA in patients with MDD was associated with more severe depressive symptoms and hopelessness [50]. A case-control study also demonstrated that hopelessness and negative life events are risk factors for SA in patients with MDD [51]. However, this present study did not identify significant associations between SA and other risk factors such as sex, family history of psychosis [50], early age of onset [52, 53], and psychosis [54]. These differences may be attributable to different social cultures, methods, and differences in the characteristics of the enrolled participants. Environmental factors have also been demonstrated to play a crucial role in SA [6, 30, 55].

However, the exact pathogenesis underlying SA in patients with MDD remains unclear. Several studies have reported gene-environment interactions in suicidal patients with MDD [6, 35, 39]. In a 22-year longitudinal gene-environment study, *HTR2A* variants (rs6561333, rs7997012, and rs1885884) were noted to interact with histories of sexual and physical abuse in patients with SA [35]. Early adversity and recent acute/chronic stress (separation, loss, interpersonal or family questions, and unemployment) have long been recognized as playing a critical role in both MDD and SA [6, 56].To the best of our knowledge, this is the first study on the interaction between *HTR2A* rs3125 and negative life events using the GMDR model in a Chinese population. Notably, this study identified a potential gene-environment interaction between *HTR2A* rs3125 and negative life events in relation to SA in patients with MDD. This finding supports the hypothesis that MDD and SA result from a combination of genetic and environmental factors.

This study is subject to several limitations. First, the study focused on the Chinese population, and the results may not apply to other countries. Second, the data used in this study were collected retrospectively. Therefore, a recall bias may have affected the accuracy of the data. However, only a step was taken to reduce the likelihood of recall bias. Third, the environmental factors and clinical features were only qualitatively analyzed as binary data. In future studies, questionnaire evaluations should be conducted to quantitatively assess these variables.

## Conclusions

A potential interaction was identified between *HTR2A* rs3125 and negative life events by GMDR. This interaction reflects a higher risk of SA when patients with MDD carrying the rs3125 GG genotype of *HTR2A* are exposed to negative life events. Therefore, this study may not only provide evidence of certain key risk factors for SA in patients with MDD but may also deepen our understanding of its pathogenesis.

### Electronic supplementary material

Below is the link to the electronic supplementary material.

Supplementary Material 1

Supplementary Material 2

Supplementary Material 3

## Data Availability

The datasets used and/or analyzed during the current study will be made available upon reasonable request. Raw data supporting the obtained results are available at the corresponding author.

## Abbreviations

MDD: Major depressive disorder
SA: Suicide attempts
HTR2A: Serotonin 2 A receptor
NOS1: Nitric oxide synthase 1
tagSNPs: Tag-single nucleotide polymorphisms
GMDR: Generalized multifactor dimensionality reduction
MDD-SA: Major depressive depression with suicide attempts
MDD-NSA: Major depressive depression without suicide attempts
HC: Healthy controls
OR: Odds ratios
CI: Confidence interval
DSM-IV: Diagnostic and Statistical Manual of Mental Disorders, Fourth Edition
HDRS-24: Hamilton Depression Rating Scale-24 items
CV: Cross-validation
NLE: Negative life events
CTH: Childhood trauma history
